# The Effect of Vitamin D3 Supplemented with PhytoSolve on Genes Involved in Implantation in an NMRI Mice Model of Polycystic Ovary Syndrome: An Experimental Study

**DOI:** 10.5935/1518-0557.20230064

**Published:** 2023

**Authors:** Sahar Hakimpour, Azam Govahi, Sahar Eghbali, Ronak Shabani, Rana Mehdizadeh, Marziyeh Ajdary, Mehdi Mehdizadeh

**Affiliations:** 1 Department of Physiology, School of Veterinary Medicine, Shiraz University, Shiraz, Iran; 2 Endometriosis Research Center, Iran University of Medical Sciences, Tehran, Iran; 3 Reproductive Sciences and Technology Research Center, Department of Anatomy, School of Medicine, Iran University of Medical Sciences, Tehran, Iran; 4 School of Dentistry, Central Tehran Branch, Islamic Azad University, Tehran, Iran

**Keywords:** vitamin D, PhytoSolve, implantation, PCOS

## Abstract

**Objective:**

Vitamin D3 has been shown to be effective in the treatment of PCOS. However, due to its poor solvability and bioavailability, effective time is delayed and dosage requirements are increased. In our previous study, we demonstrated that PhytoSolve containing VD3 is more effective than vitamin D3 alone in the treatment of PCOS. In this study, we aimed to investigate the effect of this vitamin D3 formulation on gene expression involved in implantation in patients with PCOS.

**Methods:**

To create PhytoSolve, Lipid S75, glycerol, and MCT oil were combined using a sonicator probe. Six groups, each consisting of 36 female Naval Medical Research Institute (NMRI) mice, were included in the following groups: control; sham; PCOS; PhytoSolve; PhytoSolve containing VD3; and vitamin D3. The mice were given DHEA injections to induce PCOS. After administering PhytoSolve containing VD3 and vitamin D3 by gavage for one week from the 13^th^ day of model creation, the female mice were mated and endometrial tissue was collected for analysis of *LIF, β-integrin,* and *HOXA10* proteins and genes.

**Results:**

Compared to the group receiving vitamin D3 alone, the group receiving PhytoSolve containing vitamin D3 showed a significant increase in the expression of LIF, β-integrin, and HOXA10 genes (*p*<0.05). Although there was an increase in the expression of β-integrin and HOXA10 proteins in the group given PhytoSolve containing vitamin D3 compared to the group given vitamin D3, this increase was not significant. However, the increase in LIF protein expression in the group given PhytoSolve containing vitamin D3 was significant when compared to the group given vitamin D3 (*p*<0.05).

**Conclusions:**

The use of PhytoSolve containing vitamin D3 was more effective than vitamin D3 alone. The PhytoSolve formulation might be a useful solution for medications with limited solubility and bioavailability.

## INTRODUCTION

Around the world, 6 to 12 percent of women of reproductive age suffer from polycystic ovarian syndrome (PCOS), a prevalent female endocrine disorder (Bozdag *et al.,* 2016). Some of its symptoms include menstrual irregularity, anovulatory infertility, hyperandrogenism, obesity, and metabolic disorders (Rotterdam ESHRE/ASRM-Sponsored PCOS consensus workshop group, 2004),_all of which may significantly affect a patient’s health and quality of life. However, due to the complexity of its pathophysiology, the disease’s cause remains unclear, and no effective treatment has been found as of yet (Rotterdam ESHRE/ASRM-Sponsored PCOS consensus workshop group, 2004).

Studies have shown that the endometrium may be significantly impacted by endocrine and metabolic irregularities in PCOS, which may lead to infertility, endometrial involvement, and issues such as impaired implantation processes (Donaghay & Lessey, 2007; Qiao *et al*., 2008; Govahi *et al*., 2023). Impaired uterine receptivity is one of the leading causes of spontaneous abortion in PCOS patients (Lopes *et al*., 2011).

According to studies, women with PCOS experience a decrease in HOXA-10, LIF, and β-integrin proteins during the secretory phase (Bergeron *et al*., 1988; Apparao *et al*., 2002; Cermik *et al*., 2003; Daftary *et al*., 2007). Integrins, or CAMs, are believed to be reliable indicators of endometrial receptivity and are essential in cell interaction. Integrins exhibit a sandwich-like structure in the embryonic junction (Wang & Armant, 2002; Achache & Revel, 2006; Jiang & Li, 2022; Zhang *et al*., 2022; Bajpai *et al*., 2023). Additionally, expression of LIF mRNA in the endometrium of healthy women occurs during the middle and late secretory phases of the menstrual cycle, as well as in early pregnancy (Aghajanova, 2004; Kara *et al*., 2016; Ajdary *et al*., 2020; Fukui *et al*., 2022; Bajpai *et al*., 2023). The HOXA-10 gene also has a physiological role in implantation by regulating the response of endometrial stromal cells to progesterone (Celik *et al*., 2015; Kara *et al*., 2016; 2019; Zhang *et al.*, 2022; Bajpai *et al*., 2023).

Despite the ability to select high-quality embryos during assisted reproductive procedures, implantation rates have remained low and have not significantly increased in recent years (Andersen *et al*., 2005; Amjadi *et al*., 2019; Ajdary *et al*., 2020). Uterine receptivity is a critical factor in determining the success of pregnancy and may be adjusted to increase the effectiveness of assisted reproductive technologies, particularly in gynecological conditions such as PCOS (Donaghay & Lessey, 2007). Thus, there is an increasing need for creative interventions to treat this condition effectively.

Several recent studies have described the relationship between vitamin D3 (VD3) deficiency and the progression of insulin resistance, hypertension, dyslipidemia, glucose intolerance, diabetes, obesity, and metabolic syndrome (Bahadur *et al*., 2022; Morgante *et al*., 2022; Simpson *et al*., 2022). Some studies have also highlighted the higher prevalence of VD3 deficiency in patients with PCOS and the relationship between this deficiency and the development of insulin resistance, and metabolic and endocrine conditions in these patients (Hahn *et al*., 2006; Kotsa *et al*., 2009; Wild *et al*., 2011; Thomson *et al*., 2012; Mousa *et al*., 2015; Trummer *et al*., 2018). This theory is supported by the idea that the VD3 receptor gene regulates approximately 3% of the genes in the human body, including those involved in glucose and lipid metabolism and blood pressure control (Thys-Jacobs *et al*., 1999). Additionally, VD3 adjusts the expression of 229 genes in over 30 different tissues, such as the pancreas, liver, immune cells, and ovaries (Gatti *et al*., 2016).

VD3 has low bioavailability due to its poor dissolution in water. Recent studies have shown that slow-release formulations (SRDDSs) in the drug delivery system have a favorable result in slow-release speed, reducing the number of daily doses, and improving bioavailability. Previous studies have shown that phospholipids can be used to increase the release of drugs with poor dissolution in water. In addition, phospholipids protect against drug degradation in the gastrointestinal tract (Haddadzadegan *et al*., 2022). In the PhytoSolve technique, phospholipids diffused in a very concentrated aqueous solution of carbohydrate or polyol can dissolve large amounts of lipids, steroids, terpenes, and polar lipids (Keyhanfar *et al*., 2014). In our previous study, we used VD3 loaded in the PhytoSolve formulation to treat patients with PCOS. In this study, we investigated the effect of this VD3 formulation on the expression of genes involved in implantation in patients with PCOS.

## MATERIALS AND METHODS

### Materials

Antibodies were obtained from Abcam (ab138002, ab153904, ab179471 Cambridge, United Kingdom) and TRIzol and Glycerol were prepared from Sigma Aldrich (St. Louis, MI, USA). Deionized water and SYBR Premix Ex Taq were obtained from a Milli-Q water purification system (Millipore, Burlington, MA, USA) and Applied Biosystems (Foster City, CA, USA) respectively. cDNA synthesis kits were prepared by Thermo Fisher Scientific (Waltham, MA, USA).

### Preparation of PhytoSolve containing VD3 and its physicochemical study

The preparation of PhytoSolve containing VD3 was carried out according to our previous study (Hakimpour *et al*., 2022). Briefly, 20 mg of VD3 in 2 g of medium-chain triglyceride (MCT) oil, and 0.5g of phospholipid (Lipoid S75) were homogenized in 7.5g of polyol phase at room temperature with an Ultra-Turrax homogenizer (IKA T10B, Germany). The oil phase (MCT+VD3) was slowly added to the phospholipid and polyol phase and mixed. The final mixture was sonicated with a probe sonicator (Hielscher, Germany) at 70% intensity for 10 minutes to reduce the emulsion particle size. The PhytoSolve formulation was prepared using polyol phase, and the physicochemical properties of the PhytoSolve containing VD3 were described in our previous study (Hakimpour *et al*., 2022).

### Study groups, PCOS modeling, and isolation of endometrial tissue

Thirty-six 25-day-old female NMRI laboratory mice were divided into six groups: control group, which received normal food and water without any treatment; sham group, which received sesame oil i.p for 20 days via gavage; PCOS group, which consisted of animals with polycystic ovary disease without any treatment; VD3 group, which consisted of animals with PCOS given VD3; PhytoSolve group, which consisted of animals with PCOS given the PhytoSolve formulation without VD3; and the PhytoSolve containing VD3 group, which consisted of animals with PCOS given PhytoSolve containing VD3. The treatment of the PCOS model groups was administered via gavage starting 13 days after model induction for one week.

Induction of PCOS in mice was performed according to the protocol described in our recent study (Hakimpour *et al*., 2022). Briefly, a subcutaneous injection of a mixture of dehydroepiandrosterone (DHEA), sesame oil, and 95% ethanol was administered for 20 days.

All animals were kept under standard light and dark conditions, and had free access to food and water. During the treatment period, the animals’ weight was measured every two days and vaginal smears were taken daily from 10 days after the first injection until the end of the experiment. All mice remained in the estrous cycle, and the control group was blooded on the day of estrus. Ovarian tissue was then separated and placed in 10% formalin. Additionally, blood was taken from the animals’ hearts to check fasting testosterone and insulin levels. The entire female mice were coupled with NMRI male mice. The vaginal plugs were examined in the morning after finishing the treatment, and if a plug was formed, it was considered the first day of pregnancy. Pregnant animals were euthanized with ether on day 4.5. Endometrial samples were then collected and stored at -80°C for RNA extraction.

### RNA extraction and quantitative real-time PCR (qRT-PCR)

Reagent TRIzol (Sigma-Aldrich) was used to extract total RNA from endometrial samples based on manufacturer instructions. Technical triplicates were made for every sample. In order to remove genomic DNA contamination, the entire samples were incubated with DNase I (Fermentas, St. Leon- Rot, Germany). Then, for determining RNA concentration, yield, and purity, using the A260/A280 ratio method, samples were analyzed on a spectrophotometer. Using oligo dT primers (Metabion, Martinsried, Germany) and the SuperScript First-Strand Synthesis System (200 U/ ml, Invitrogen), total RNA was reverse transcribed. One converse transcription control carried out under the aforementioned conditions without SuperScript II enzyme was utilized in every PCR cycle. In primary tests, using a conventional PCR protocol (Invitrogen), the primer pairs were tested, and the products run on agarose gel were limited to a single band of the expected size. Table 1 presents the gene-specific primers used. Negative controls without cDNA were included in all experiments. qPCR reactions were performed as *previously described* (Ajdary *et al.,* 2020). To ensure the removal of contaminants or primer dimers, PCR reactions’ melting curves were monitored. Using the cDNA’s logarithmic dilution series, standard curves were obtained for every gene. Normalization of the threshold cycle values was based on the threshold value of human GAPDH. Using the comparative CT method, the data of qPCR were analyzed (Ajdary *et al.,* 2021; Govahi *et al.,* 2023).

**Table 1:** The sequence of the primers used in this study.

Gene		Sequence (5′- > 3′)	Length	Tm
**Leukemia inhibitory factor (*LIF*)**	Forward	TTTCCAGGTACTCACTGCACTC	22	59.96
	Reverse	TCTCAGACCAACACCCTCATTG	22	59.96
**Homeobox gene 10 (*HOXA10*)**	Forward	TGTTTAATCAGGGAGTCCAGGC	22	60.03
	Reverse	TTTTTCAACCAGCCAGGTCAAG	22	59.57
**Integrin beta-1(*β-Integrin*)**	Forward	GCAACCTTCGGATTGGCTTT	20	58.00
	Reverse	GGCAAGCAGGCATTCTTCAT	20	58.00
**Glyceraldehyde 3-phosphate dehydrogenase (GAPDH)**	Forward	CAAGATCATTGCTCCTCCTG	20	58.40
	Reverse	ATCCACATCTGCTGGAAGG	19	57.30

### Immunohistochemistry (IHC)

IHC staining was performed to determine *LIF, HOXA10, β-integrin* proteins. IHC DAB staining was used to observe *LIF, HOXA10, and β-integrin* protein expression. The mice underwent transcardiac perfusion with 4% paraformaldehyde in phosphate buffer and then slaughtered. The uterus of each mouse was removed and postfixed overnight. Then, they were dehydrated in an ascending alcohol series, rinsed with xylene, and embedded in paraffin. Then the blocks were divided into 5 µm sections. To prepare the slides for immunostaining analysis, the antigen retrieval process involved soaking them in a citrate buffer for 10 minutes. Triton X-100 0.3% (30 minutes) and normal goat serum (1 hour) were used to permeabilize and block the slides, respectively. The sections were incubated overnight at 4°C with primary antibodies for *HOXA10* (rabbit an*ti-HOXA10* (1:100, Abcam, Cambridge, United Kingdom)), *LIF* (rabbit anti-*LIF* (1:100, Abcam, Cambridge, United Kingdom)) and *β-Integrin* (rabbit anti- *β-Integrin* (1:100, Abcam, Cambridge, United Kingdom)). The slices were then incubated in secondary antibody at 37°C for 90 min. Peroxidase-conjugated secondary antibodies were used for chromogenic detection by oxidizing 3,3′-Diaminobenzidin (1:200, ab205718) according to manufacturer instructions. *Hematoxylin* was used to stain nuclei in IHC.

### Statistical analysis

The results were explained using mean and standard error. Statistical analysis was performed using one-way ANOVA and the internal Tukey’s multiple comparisons test as post hoc. A *p*-value of 0.05 or less was considered significant.

### Ethics approval

All studies were executed in accordance with the Guide for the Care and Use of Laboratory Animals (National Institute of Health Publication No. 80-23, revised 1996) and were approved by the Research and Ethics Committee at Iran University of Medical Sciences (IR.IUMS.REC. 1399.1069), Tehran, Iran.

## RESULTS

Assessment of the physicochemical properties of PhytoSolve containing VD3

The average particle size in the PhytoSolve formulation was 71.10±1.100 nm, and after 120 hours, 91.5% of the vitamin D3 had been released from it (Hakimpour *et al.,* 2022).

### PCOS model confirmation

PCOS model confirmation was done based on the method presented in our previous study and based on body weight, number of follicles, and insulin and testosterone levels (Hakimpour *et al.,* 2022).

### Evaluation of HOXA10, β-integrin, and LIF gene expression in endometrial tissue by qRT-PCR

**Figure 1:**
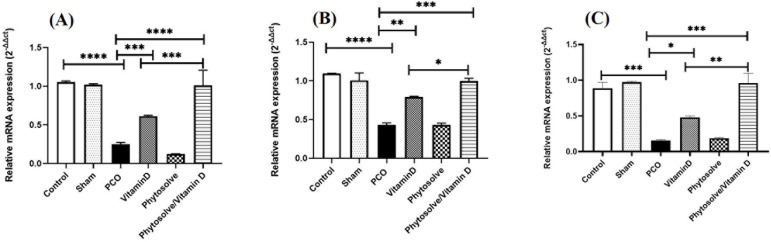
Gene expression *HOXA10* (A), *β-Integrin* (B), *LIF* (C) in endometrium from PCOS mice. The mRNA assay were performed by qRT-PCR. Data was presented as Mean ± SD (n=6 per every group). Internal control was done by β-actin. *p<0.05, **p<0.01, ***p<0.001, and ****p<0.0001.

According to Figure 1, the group with PCOS had lower levels of *HOXA10, β-integrin,* and *LIF* gene expression than the other groups, and this difference from the control group was significant (*p*<0.001, *p*<0.0001). Also, the *HOXA10, β-integrin,* and *LIF* gene expression in the VD3 and VD3-containing PhytoSolve groups increased significantly in comparison to the group with PCOS (*p*<0.05, *p*<0.01, *p*<0.001). In addition to the expression of the *HOXA10, β-integrin* and *LIF* genes in the VD3-containing PhytoSolve group were significantly increased in comparison to the VD3 group (*p*<0.05, *p*<0.01).

### Evaluation of HOXA10, LIF, and β-integrin protein levels in endometrial tissue by immunohistochemistry

**Figure 2:**
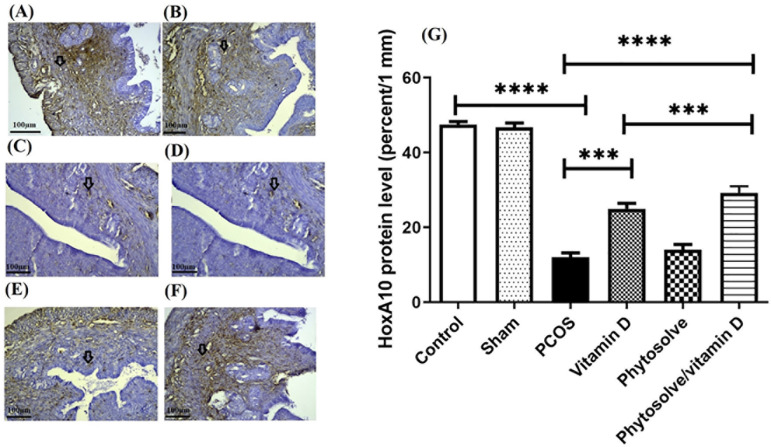
The expression of HOXA10 protein in endometrium from PCOS mice; The assay was performed by Immunohistochemistry technique. Data was presented as Mean ± SD (n=6 per every group). Internal control was done by β-actin. ***p<0.001, and ****p<0.0001. A: Control, B: Sham, C: PCOS, D: Vitamin D, E: Phytosolve, F: Phytosolve/ Vitamin D.

According to Figure 2, the level of *HOXA10* protein expression in the group with PCOS decreased significantly when compared to the control group (*p*<0.0001). The increase in *HOXA10* protein expression between the group receiving VD3-containing PhytoSolve and the group with PCOS was also significant (*p*<0.0001). The increase in *HOXA10* protein expression between the group receiving VD3 and the group with PCOS was also significant (*p*<0.001). The increase in *HOXA10* protein expression in the group receiving VD3-containing PhytoSolve increased compared to the vitamin D3 group, although not significantly.

**Figure 3:**
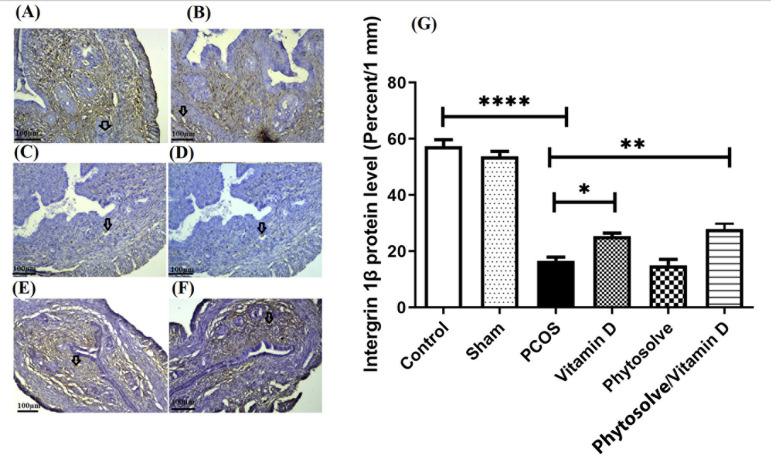
The expression of β-Integrin protein in endometrium from PCOS mice; The assay was performed by Immunohistochemistry technique. Data was presented as Mean ± SD (n=6 per every group). Internal control was done by β-actin. *p<0.05, **p<0.01, ***p<0.001 and ****p<0.0001. A: Control, B: Sham, C: PCOS, D: Vitamin D, E: Phytosolve, F: Phytosolve/ Vitamin D.

Figure 3 shows that the level of *β-integrin* protein expression in the group with PCOS decreased significantly compared to the control group (*p*<0.0001). The increase in *β-integrin* protein expression between the group receiving VD3-containing PhytoSolve and the group with PCOS was also significant (*p*<0.01). The increase in *β-integrin* protein expression between the group receiving VD3 and the group with PCOS was also significant (*p*<0.05). The increase in *β-integrin* protein expression in the group receiving VD3-containing PhytoSolve increased against the VD3 group, although not significantly.

**Figure 4:**
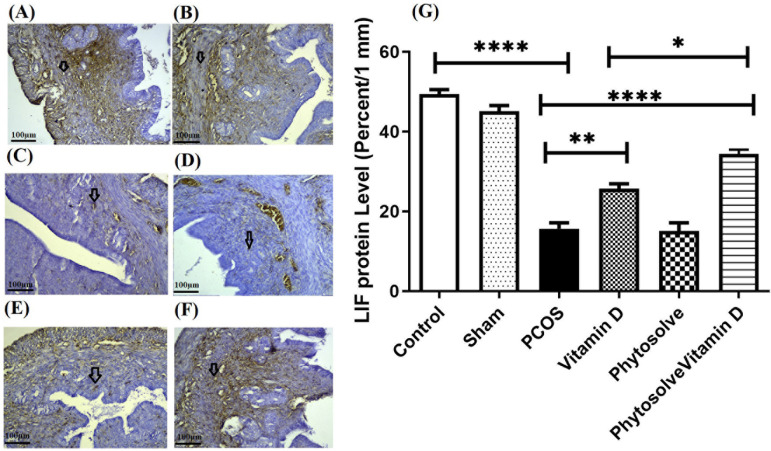
The expression of *LIF* protein in endometrium from PCOS mice; The assay was performed by Immunohistochemistry technique. Data was presented as Mean ± SD (n=6 per every group). Internal control was done by β-actin. *p<0.05, **p<0.01, and ****p<0.0001. A: Control, B: Sham, C: PCOS, D: Vitamin D, E: Phytosolve, F: Phytosolve/ Vitamin D.

Figure 4 shows that the expression level of *LIF* protein in the group with PCOS decreased significantly compared to controls (*p*<0.0001). The increase in *LIF* protein expression between the group receiving VD3-containing PhytoSolve and the group with PCOS was also significant (*p*<0.0001). The increase in *LIF* protein expression between the group receiving VD3 and the group with PCOS was also significant (*p*<0.01). There was a significant increase in *LIF* protein expression in the group receiving VD3-containing PhytoSolve against the VD3 group (*p*<0.05).

## DISCUSSION

The results of this study showed that the gene expression of *LIF, β-integrin,* and *HOXA10* in the endometrial tissue of mice with PCOS in the PhytoSolve containing VD3 group significantly increased compared to the VD3 group. Also, the expression of proteins *β-integrin* and *HOXA10* increased in the PhytoSolve containing vitamin D3 group against the vitamin D3 group, although not significantly. The increase of *LIF* protein in the PhytoSolve-containing vitamin D3 group was significant against the vitamin D3 group.

Studies have reported that the formulation of drugs based on lipids is a strategy that has produced successful results. The use of drug-phospholipid-oil complex in a liquid system was first described by Wang *et al.* (2008). They prepared a formulation containing MCT, the herbal medicinal compound Hydroxysafflor yellow A, phospholipid, and surfactant, and reported better kinetic parameters than the aqueous solution of the drug (Wang *et al.,* 2008). Further research has shown that drug entrapment in the phospholipid layer reduces its bacterial and enzymatic degradation during the absorption process (Sun *et al.,* 2013). The effect of lipids on the bioavailability of orally administered drugs is very complex, because they can change the biopharmaceutics properties of drugs by many mechanisms, including reducing the rate of gastric emptying, increasing the rate of solubility in intestinal fluid, and enhancement of lymphatic transport of hydrophobic drugs through lipoprotein formation. Factors such as the length of the triglyceride chain, the degree of saturation, and the volume of prescribed lipid affect the absorption and blood-lymph distribution of the drug, which improve the absorption of the drug in the target tissue.

In this study, the effect of VD3-containing PhytoSolve on endometrial receptivity markers was investigated for the first time. Results showed that *LIF, β-Integrin,* and *HOXA10* were highly expressed in the VD3-containing PhytoSolve group against the VD3 group. Therefore, the hypothesis was raised that encapsulating lipophilic drugs in nanometer-sized phospholipids might improve the pharmacological properties of the drugs and increase their therapeutic effect (Webb *et al*., 2012). During an in vivo study in rats, Khani & Keyhanfar (2014) showed that PhytoSolve and Phosal-based formulations of mebodipine are more available compared to oil solutions. Also, Wajda *et al*. (2007) increased the bioavailability of coenzyme Q10 and vitamin E through the use of PhytoSolve (Wajda *et al*., 2007). One of the components of PhytoSolve is pure phospholipids. S75 is used to produce a wide range of drug delivery systems to improve dissolution, stability, and delivery of the active pharmaceutical ingredient to the target site, such as mixed micelles, different types of liposomes, SLN, and more (Bocca *et al*., 2015). In our previous and recent study, S75 lipoid helped to treat PCOS (Hakimpour *et al*., 2022) and increase implantation markers. Previous reports showed that drug delivery based on nanoparticles reduces IC50 and improves pharmacological activity (van Hoogevest, 2017). Other studies that formulated drugs with phosal 50PG showed that this model increased the level of the active substance in the blood and improved therapeutic effects (Fricker *et al*., 2010). In general, phospholipids increase the dissolution of the active substance, maintain drug dissolution in the GI tract, and improve drug absorption and bioavailability (Fricker *et al*., 2010).

Vitamin D deficiency can cause a decrease in 1,25-OHD, insulin secretion, insulin receptor and an increase in insulin sensitivity, inflammation and oxidative stress. Vitamin D deficiency can also result in a decrease in SHBG and an increase in testosterone levels, leading to hyper-androgenism, hirsutism and acne. Additionally, vitamin D deficiency can disrupt calcium regulation, leading to halted follicular growth and lack of ovulation and infertility (Morgante *et al*., 2022).

The results of our study also showed that VD3-containing PhytoSolve improved the bioavailability of VD3 and had a more positive effect on endometrial receptivity and expression of genes involved in implantation than free VD3. The population included in this study consisted of only 36 mice; further studies with larger populations are needed to determine whether the results observed in this study are valid. A population of limited size was one of the limitations of this study.

## CONCLUSION

Based on our results, VD3 with PhytoSolve was more successful at increasing the expression of genes involved in implantation in the PCOS model than vitamin D3 in suspension. Therefore, the Phytosolve formula is a reliable drug delivery method for medications with limited solubility and bioavailability.
